# Co-design of a digital dietary intervention for adults at risk of type 2 diabetes

**DOI:** 10.1186/s12889-021-12102-y

**Published:** 2021-11-11

**Authors:** Brenda S. J. Tay, Sarah M. Edney, Grant D. Brinkworth, David N. Cox, Bonnie Wiggins, Aaron Davis, Ian Gwilt, Annemien Haveman-Nies, Jillian C. Ryan

**Affiliations:** 1 Nutrition & Dietetics, College of Nursing & Health Sciences, Flinders University, GPO Box 2100, Adelaide, South Australia 5001 Australia; 2 Physical Activity and Nutrition Determinants in Asia (PANDA), Saw Swee Hock School of Public Health, National University of Singapore, Singapore, Singapore; 3 Commonwealth Scientific and Industrial Research Organisation (CSIRO) – Health and Biosecurity, 11 Julius Avenue, North Ryde, NSW 2113 Australia; 4 Commonwealth Scientific and Industrial Research Organisation (CSIRO) – Health and Biosecurity, 13 Kintore Avenue, Adelaide, SA 5000 Australia; 5 UniSA Creative, University of South Australia, Adelaide, Australia; 6 Consumption and Healthy Lifestyles, Wageningen University and Research, Wageningen, The Netherlands

**Keywords:** Co-design, Participatory research, Digital dietary intervention, Pre-diabetes, Type 2 diabetes, Health behaviour

## Abstract

**Background:**

Co-design has the potential to create interventions that lead to sustainable health behaviour change. Evidence suggests application of co-design in various health domains has been growing; however, few public-facing digital interventions have been co-designed to specifically address the needs of adults at risk of Type 2 diabetes (T2D). This study aims to: (1) co-design, with key stakeholders, a digital dietary intervention to promote health behaviour change among adults at risk of T2D, and (2) evaluate the co-design process involved in developing the intervention prototype.

**Methods:**

The co-design study was based on a partnership between nutrition researchers and designers experienced in co-design for health. Potential end-users (patients and health professionals) were recruited from an earlier stage of the study. Three online workshops were conducted to develop and review prototypes of an app for people at risk of T2D. Themes were inductively defined and aligned with persuasive design (PD) principles used to inform ideal app features and characteristics.

**Results:**

Participants were predominantly female (range 58–100%), aged 38 to 63 years (median age = 59 years), consisting of a total of 20 end-users and four experts. Participants expressed the need for information from credible sources and to provide effective strategies to overcome social and environmental influences on eating behaviours. Preferred app features included tailoring to the individual’s unique characteristics, ability to track and monitor dietary behaviour, and tools to facilitate *controlled* social connectivity. Relevant persuasive design principles included *social support*, *reduction (reducing effort needed to reach target behaviour)*, *tunnelling (guiding users through a process that leads to target behaviour)*, *praise*, *rewards*, and *self-monitoring*. The most preferred prototype was the *Choices* concept, which focusses on the users’ journey of health behaviour change and recognises progress, successes, and failures in a supportive and encouraging manner. The workshops were rated successful, and feedback was positive.

**Conclusions:**

The study’s co-design methods were successful in developing a functionally appealing and relevant digital health promotion intervention. Continuous engagement with stakeholders such as designers and end-users is needed to further develop a working prototype for testing.

**Supplementary Information:**

The online version contains supplementary material available at 10.1186/s12889-021-12102-y.

## Introduction

Type 2 diabetes (T2D) continues to be a leading cause of premature death and disability worldwide [1]. Whilst T2D risk is influenced by a multitude of factors such as socioeconomic characteristics, geographical location, health literacy and culture [2], poor dietary behaviour and physical inactivity are primary modifiable risk factors [3]. This public health problem is escalating as changing food systems increasingly promote nutritionally imbalanced dietary intakes and sedentary lifestyles with low physical activity levels [4–6].

Digital health technologies including the use of smartphones, websites and/or text messaging to deliver digital health interventions continue to gain traction in chronic care management seeking to improve the translation of health advice into the community [7]. The potential of digital health interventions as scalable, cost-effective tools to improve health and healthcare delivery is well-established [7]. Furthermore, in person-centred dietetic practice, the use of digital technologies to deliver interventions that promote individual behaviour change and habit formation is increasingly recognised as an innovative approach to support chronic disease management [8].

Despite the potential benefits of digital health technologies, adoption by health behaviour change interventions can be slow, and usage is not sustained long after initial implementation [7, 9–12]. This is due in large part to low user engagement in the intervention development process, impeding the long-term impacts of digital health solutions.

Co-design is a participatory approach to designing products or solutions that often leads to sustainable usage [13], offering potential design and development application for translational digital health tools targeting chronic disease including T2D. Co-design considers users’ needs, desires, characteristics and abilities throughout the design process [13], that in this case address the central role of individual behaviour in chronic disease management [14]. In research contexts, co-design is grounded in participatory action research (PAR) principles, a “bottom-up” approach recognised to have significant advantages in translational health research. A key characteristic of PAR is involvement of researchers collaborating with end-users and key stakeholders in the process of research, policy and program development to produce outputs highly relevant to community needs [15]. When used in a healthcare setting, co-design has the potential to integrate end-user and healthcare worker experiences’ (as well as other stakeholders), to collaboratively explore solutions to local problems [16]. *Making, or thinking while doing* is considered to be key within co-design practice and occurs through three approaches: probes (materials used to evoke users’ experiences and behaviours that inspire design), generative toolkits (used to produce artefacts and depictions of their use which are then analysed to identify patterns), and prototypes (tangible manifestations of ideas or concepts) [17]. The act of making is creative, processes based and involves both construction and the transformation of meaning from these activities [17]. The making process can also be described as a divergent process, with a focus on increasing knowledge and the generation of new ideas [18]. This is often paired with convergent processes of co-analysis where stakeholders collaboratively work through the generated information to identify and prioritise key elements [18]. Co-design processes are varied and are adapted to suit the needs of the participants, but typically involve iterative or multiple stages of engagement.

In Sanders & Stappers [17] model of co-design this is mapped through four main phases: *pre-design*, *generative*, *evaluative* and *post-design* [17]. The focus of pre-design is the broader context of experience, whereas post-design studies people’s actual experience of the product, service, or space [17]. The generative phase (stage 2) guides decision-making to identify design opportunities, and the evaluative phase covers the iterative design process whereby prototypes are tested and refined with stakeholders [17]. In the British Design Council’s Double Diamond Design Process model, there are also four phases which blend divergent and convergent approaches: discover, define, develop, and deliver [18]. The discover phase aligns with Sanders & Stappers’ [17] pre-design phase, while the define phase is a specific process of revisiting and prioritising the main challenges that have been uncovered and that should be taken forward into the generative develop phase [18]. The generative, evaluative, and post-design stages of Sanders & Stappers’ model roughly align with the develop and deliver phases of the Double Diamond as a cyclical and iterative process based on prototype development, testing and iteration [17, 18].

Co-design offers a promising approach to design public health interventions in line with person-centred practice; however, although its application in various health research domains is growing, examples of well-documented co-design developing digital dietary interventions as a main research outcome are limited. Previous applications of co-design include the development and evaluation of interventions targeting smaller meal portions [19] and physical activity promotion [20]. Other examples have targeted specific populations, including older adults with age-related macular degeneration [21] and Indigenous communities [22]. Whilst these are promising examples, further research is needed to demonstrate how co-design can be conducted and reported in a rigorous manner and its value realised for different health applications. To our knowledge, there are currently no public facing digital interventions, for example smartphone-based apps, which have been co-designed to specifically address the needs of adults at risk of T2D [23].

## This study

The co-design process in this study is built upon the findings of a Delphi study which explored the health needs, contexts and experiences of key stakeholders with regard to diabetes prevention [24]. The Delphi study therefore provided a baseline from which the co-design process commenced. Participants in the Delphi study included 38 Australians with pre-diabetes or T2D and 38 professional stakeholders including dietitians, credentialed diabetes educators, nurses, medical doctors, research scientists, and exercise physiologists. The Delphi study identified physical activity, diet, and mental health as priority areas for intervention, with access to healthcare services and resources to support health literacy, access to self-monitoring technologies, online support networks, and success stories also identified as crucial for facilitating behaviour change.

The co-design process engaged with these topics, exploring them in detail with a group of ‘lived experience’ stakeholders working toward design-based solutions that could address each topic. Since this study’s aim was to test the application of the co-design approach in intervention development, this paper will only focus on the diet aspects of T2D management. The co-design study had two objectives: (1) to co-design, with end-users, a digital dietary intervention to promote health behaviour change among adults at risk of T2D, and (2) to evaluate the co-design process involved in developing a digital dietary intervention prototype.

## Methods

The study received ethics approval from the Commonwealth Scientific and Industrial Research Organisation (CSIRO; approval number 2019_102_LR) and has been reported according to the Consolidated Criteria for Reporting Qualitative Research (COREQ) checklist [25].

### Study design

The study design is qualitative research using a co-design (participatory) framework [17].

### Participants

Individuals who participated in the Delphi study were invited to join the co-design study if they met the following eligibility criteria: (1) living in Australia, (2) aged 18 years or older, (3) end-users: self-identifying as having pre-diabetes or at risk of T2D or having T2D at present or any time in the past (as indicated by the answer to the question – has your doctor told you that you have or had pre-diabetes or T2D?), *or* professional/clinical experts: having at least 2 years of diabetes-related work experience, and (4) having access to an internet-connected device (e.g., computer, tablet, smartphone).

### Co-design team

The co-design project team was comprised of a research team including two behavioural scientists and a Nutrition & Dietetics expert, and a design team led by two academic designers with extensive experience in co-design for health who were assisted by four other designers as facilitators.

### Sampling and recruitment

Three recruitment rounds (one per workshop) took place between September and October 2020. Convenience and purposeful sampling were used, i.e., participants from the Delphi study were invited via email. Interested participants completed an online survey (Alchemer LLC, Boulder, CO, USA) including informed consent and demographics. Guided by previous co-design conventions [26], the target sample size was 20–25 participants per workshop. Participants received an honorarium in the form of an e-gift card ($20.00 AUD) for each workshop attended. Participants could attend as many or as few workshops as desired. It is important to note that even though the focus of the workshops was design of a T2D prevention intervention, people with pre-diabetes or T2D were eligible to participate in the study. This is because people with T2D are likely to have attempted health behaviour change before and this lived experience, providing valuable insights regarding the challenges and successes related to this.

### Co-design workshops

Due to the COVID-19 pandemic, the co-design workshops were conducted online. Three online workshops were held over 7 weeks (September–October 2020). Three online platforms were utilised: a video conferencing service, Zoom (Zoom Video Communications Inc., San Jose, CA, USA), a web-based visual collaboration platform, Miro (Miro, San Francisco, CA, USA), and interactive presentation software, Mentimeter (Mentimeter, Stockholm, SE, EU). The design team facilitated the workshops while the CSIRO researchers acted as co-facilitators and observed the workshops.

**Fig. 1:**
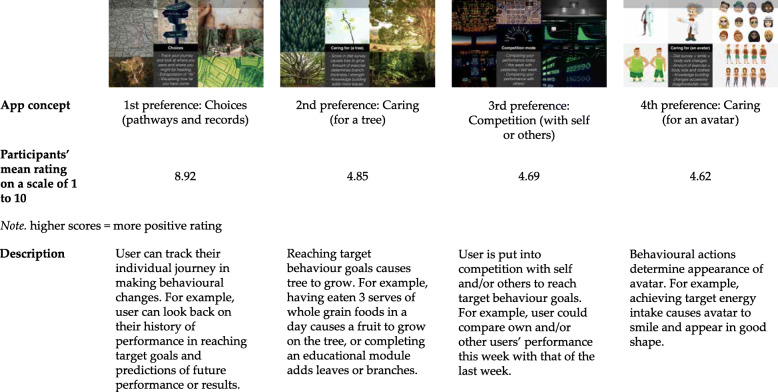
Four App Concepts Ranked According to Participants’ Preferences

Each workshop was 150 min in duration. Participants were assigned to groups of six (maximum), with a balance of scientific/clinical experts and end-users in each group. Workshops commenced with an ice breaker aimed at building rapport between participants and facilitators, and to stimulate creative thinking prior to subsequent research activities. Each workshop was then structured around four to five activities that were underpinned by content analysis. Details of workshop content is included below:

- Workshop 1 Discover: In this workshop the findings from the Delphi study were used to guide the development of two main activities. The first activity extended the findings from the Delphi study by using a collaging exercise to translate abstract concepts such as “simple and easy to understand” into practical examples. This process allowed the team to uncover features of information that the potential end-users present in the workshop found “simple and easy to understand” as well as those that made information “complex and hard to understand”. The second activity focused on challenging the assumption that access to information, self-monitoring, and online support and success stories would lead to behaviour change. This activity asked participants to explore their personal mobile phones and to self-evaluate the kinds of apps they had installed, as well as the frequency of their use. Upon sharing with their small groups, the importance of driving engagement rather than the passive provision of a service emerged as being of critical importance.
- Workshop 2 Define: This workshop focused on convergent analysis processes to identify key pieces of information that should be carried forward in the project. It used a co-analysis process to review the design researchers’ interpretations of the findings from Workshop 1, checking assumptions and gaps to ensure the findings were representative of the end-users’ contributions. The workshop then focused on identifying the specific kinds of information that users of a T2D app may find useful. An anonymous form of contribution was used to create a safe space for participants to respond to questions such as “what are people too embarrassed to ask?”. The collated responses from this activity were then taken back into the collaborative workshop space to explore who this information should and should not be delivered by, and specifically which types of information people would feel comfortable receiving from an app or digital source. Following Workshop 2, the design academics developed four app concepts or prototypes (Fig. 1) that were used in Workshop 3 for the purpose of soliciting feedback on potential app designs.
- Workshop 3 Develop: In the final co-design workshop of this part of the project, abstraction was re-introduced to encourage participants to think creatively and to identify new opportunities. The workshop began by presenting participants with abstract ‘app icons’ and asking them to collaboratively describe what features they represented and whether they should be included in a T2D app or not. These features were then carried forward into a Job Story template to explore how, when, where, and by whom the feature may be used, eliciting further creative reflection on how a T2D app may be used. The final part of this workshop presented the group with a series of mock-up prototypes of the app. Throughout the workshop series, process evaluation data was collected through team discussions and de-briefing sessions, as well as via online feedback surveys from participants.

### Participatory process

We employed a number of strategies to encourage and enable achieve active participation by all participants. These included:

- trained facilitators with at least 2 assistant facilitators available to help with facilitation or assist individual participants as required
- no assumed domain knowledge and minimal technical skills required to participate, beyond joining the online workshop via freely available software (Zoom)
- online workshops that can be joined from home and entered/exited freely
- ability for participants to direct message or speak aloud their ideas if unable to access software platforms used like Miro
- icebreaking activities that introduced participants to each other in a hierarchy-disrupting way (ensuring contributions of equal depth and complexity could be made by all participants) and modelled the process of contributing to the online collaboration platform.
- providing practice activities and scheduling an online drop-in session in advance of the workshops to familiarise participants with the online collaboration platform and to meet a member of the research team.
- using breakout small-group discussions (up to 5 people) as well as anonymous contribution opportunities to ensure participants felt safe to contribute their thoughts and ideas.

### Data analysis

Inductive, iterative analysis was used to analyse the workshop activity content generated by participants. The data analysis involved 1st-order categories, further grouped to form 2nd-order themes, then distilled into aggregate dimensions [27]. Data consisting of digital sticky notes, responses to Mentimeter questions and discussions were coded in the first order analysis. In creating sub-themes (2nd-order themes), codes were attributed to relevant domains under the Theoretical Domains Framework (TDF), an integrated theoretical framework for identifying influences on behaviour [28]. For each major theme (aggregate dimensions), sub-themes were used to inform principles of persuasive design (PD), a rigorous method of designing app components that takes into consideration users’ perceptions and interpretations of app content and visuals [29, 30]. Example implementation recommendations were generated through team discussion and are intended to illustrate a few examples potential implementation pathway rather than an exhaustive list.

Data analysis was managed using Microsoft Word. Data from the first workshop were analysed independently by two members of the authorship team with any discrepancies resolved through discussion. Data from the second and third workshops were analysed by the Nutrition and Dietetics expert independently with support from the two behavioural scientists for feedback and refinement.

## Results

### Participants

Across all workshops, participants were mainly female [Workshop 1: *n* = 10 (83%); Workshop 2: *n* = 9 (100%); Workshop 3: *n* = 7 (58%)], aged 38 to 63 years old (median age = 59 years), and consisted of 20 end-users and four experts in total. Distribution of end-user and scientific/clinical expert stakeholders was relatively balanced across the workshops with a range of 7–11 end-users and 1–4 scientific/clinical experts attending each workshop. Participants in Workshop 1 tended to have completed a higher level of education compared to participants in Workshop 3 (8/12 vs 2/12 participants had university degrees). There was also large variation in the diabetes status of participants between the workshops such that in Workshop 1 only 1/12 participants reported having T2D, compared with majority of participants (8/12) in Workshop 3.

Participant characteristics are shown in Table 1.

**Table 1:** Characteristics of Co-Design Workshop Participants

Characteristic		Workshop 1	Workshop 2	Workshop 3	Total ^a^
Stakeholder type, n	End-user	8	7	11	20
	Scientific/clinical experts	4	2	1	4
	Total	12	9	12	33
Sex, n	Female	10	9	7	17
Age (years)	Median	55	55	59.5	59
	Range	38–63	38–61	40–62	38–63
Highest level of education attained, n	Year 12 or equivalent	1	2	5	5
	Trade certificate or diploma	3	3	5	9
	University degree (e.g., bachelor’s degree)	3	1	1	4
	Postgraduate university degree	5	3	1	6
Cultural identity, n	Australian	10	8	9	19
	Chinese	1	1	0	1
	English	1	0	1	2
	New Zealand	1	0	0	1
	South African	0	0	1	1
	Irish	0	0	1	1
	Scottish	0	0	1	1
Health status, n	Pre-diabetes	3	2	2	5
	Type 2 diabetes	1	1	8	9
	None of the above	5	4	1	6
	Unsure	1	1	1	1
	Did not indicate in survey	2	1	0	3
	Has other serious health conditions	1	1	0	2
Index of disadvantage SEIFA ^b^ score by postal code residence, n	1–25 (percentile)	*n* = 4	*n* = 2	*n* = 3	*n* = 6
	26–50 (percentile)	*n* = 3	*n* = 3	*n* = 4	*n* = 8
	51–75 (percentile)	*n* = 0	*n* = 2	*n* = 1	*n* = 2
	76–100 (percentile)	*n* = 5	*n* = 2	*n* = 4	*n* = 8

### Desired app features and characteristics related to intervention function and information provision

Data from the workshops that identified features related to desired education content and intervention design, including suggested persuasive design principles that could be implemented are summarised in Tables 2 and 3, respectively.

**Table 2:** Educational topics that should be incorporated in an app for adults at risk of T2D

Workshop findings	Implementation recommendations—Persuasive design principles^a^ and implementation examples related to workshop findings
**Theme:** T2D Stigma. People are influenced by negative connotations attached to people with T2D. **TDF Domain:** Social influences **Example quote:** “… i thought it was just fat lazy people who got type 2”	**Trustworthiness:** App should provide information that is genuine and non-discriminatory. For example: app uses language that does not perpetuate stigmatization of people with T2D, app recognises individual’s journey and focuses on individual goals.
**Theme:** Impacts of disease. People require support to deal with the social, financial, and psychological impacts of T2D. **TDF Domain:** Emotion **Example quote/s:** “How do I go about eating over at friends or out at restaurants, so I don’t seem different?”, “That other people are going through the same thing and how to find those people”, “Am I going to die?”	**Normative influence:** App should enable users to gather with other users who have similar goals and make them feel norms. Examples include mechanisms to create groups or features a community forum for peer support. **Praise:** App should provide feedback information based on user’s behaviours using words, images, or sounds. Examples include automated text-messages to encourage, motivate, and empower users to reach individual goals. **Tunnelling:** App should provide action pathways that facilitate reaching target behaviour. Examples: App provides information or access to professional dietary advice/individualised dietary education and other health services.
**Theme:** Individual, social, and environmental determinants People require additional support to manage their condition in the context of these determinants. **TDF Domain:** Environmental context and resources **Example quote/s:** “I receive food via a local food bank program to help get me by since my job has stood me down. It’s all white bread, pasta and potatoes … maybe 3 leaves of silver beet and some cans of things. What/how do make this work?”	**Reduction:** App should reduce effort that users need to adopt target behaviour. For example, app provides information on practical and budget-friendly dietary strategies. **Tunnelling:** App should provide action pathways that facilitate reaching target behaviour. For example, app presents tools for adopting behaviour such as menu plans and shopping lists in a sequential pattern.
**Theme:** Credible sources of information People want to receive information from credible and reliable sources. **TDF Domain:** Reinforcement **Example quote/s:** (Participants do not wish to get answers/information from) “Anyone unskilled in dietary advice or who jumps to blame the person”	**Real-world feel:** App should provide details of the organisation and/or people involved in delivering the app’s content and services. For example, app allows users to reach specific people with expertise (e.g., healthcare professionals) through sending feedback or asking questions. **Authority:** App should refer to people or organisations with authority. For example, app quotes/references authorities e.g., government health board, global health authorities. **Third-party endorsements:** App should show endorsements from reputable sources, for example, app displays logos of reputable partners and stakeholders. **Verifiability:** App should enable users to verify the accuracy of content by redirecting them to external sources, for example, app provides links to other verified sources where content is displayed.

^a^PD principles originally described by Oinas-Kukkonen & Harjumaa (2009)

**Table 3:** Features that should be incorporated in an app for adults at risk of T2D

Workshop findings	Implementation recommendations—Persuasive design principles^a^ and implementation examples related to workshop findings
**Theme:** Individual, social and environmental determinants **TDF domain:** Environmental context and resources **Example quote:** “Budget menu planner, with recipes, so if you’re broke you can still eat for good T2 management …”	**Reduction:** App should reduce effort that users need to adopt target behaviour. For example, app lists budget-friendly healthy food options at restaurants and on grocery shopping websites.
**Theme:** Access to healthcare services **TDF domain:** Environmental context and resources **Example quote:** “Keep me on track, help me understand my processes, determine exactly what is unfolding instead of guessing … from which community or doctors may be able to help me.”	**Tunnelling:** App should provide action pathways that facilitate reaching target behaviour. For example, app offers information about available professional healthcare services.
**Theme:** Social connections **TDF domain:** Social influences **Example quote:** “Chat groups with break out facilities so people can connect and support each other if they want”	**Social learning:** App should enable users to view other users who are adopting similar target behaviours. Users can connect with other users via chat groups, a community forum, Facebook, or other social media platforms. **Social facilitation:** App should enable users to discern other users who are adopting similar behaviours. User can have video conference calls with healthcare professionals.
**Theme:** Tracking/monitoring progress **TDF domain:** Behavioural regulation **Example quote:** “… Identifies that you have gone over your carbs for the day, for example”	**Self-monitoring:** App should enable users to track their status or performance. For example, app presents a user’s diet record.
**Theme:** Unique journey **TDF domain:** Social/professional role and identity **Example quote:** “Analysis of the menu items linked to personalisation of the app for you and utilising historical data”	**Personalisation:** App should offer personalised content and services. Users can be guided to make healthier food choices based on ability of app to analyse nutritional information of food and keep a record of user’s progress.
**Theme:** Behavioural consequences **TDF domain:** Reinforcement **Sub-theme:** Positive reinforcement **Example quotes:** “Assist/force exercise habits”, “Award points for success”	**Praise:** App should provide feedback information based on user’s behaviours using words, images, or sounds. For example, app sends automated text-messages, audio, or visual notification to encourage, motivate, and empower users to reach target behavioural goals. **Rewards:** App should provide users with virtual rewards as credit for performing target behaviour. For example, app alters media items such as sounds to reward user’s performance; app gives users reward points for achieving individual goals.

^a^PD principles originally described by Oinas-Kukkonen & Harjumaa (2009)

Workshop 1 identified a high proportion of the apps that participants frequently used in general were social media apps or apps facilitating interaction and communication. This can be attributed to the *social influences* domain under the TDF. Apps that had large user bases, make life easier to manage (e.g. email, online shopping), exercise or fitness tracking apps, and apps with short form content such as news or education were also popular. The second theme, information and education, informs how the information provided through the app should be framed. We first inquired about information that is unappealing or frustrating to participants, which including information that is complex and hard to understand, for which there is ‘no right answer’ (abstract) or uses scientific or technical language. When discussing the complexity of language to be used in the app, one participant indicated “If there is too much it is overloading [and] too conflicting”. An emergent theme in this workshop was a significant volume of discussion about seemingly contradictory information, and a perception that there should be a “right answer” or “single truth”. Suggestions to overcome this complexity included to build information into manageable chunks, drawing the connections between different information, and connecting health or medical facts with the everyday experience are important principles for designing app-based education content. This theme persisted through all three workshops.

Workshop 2 identified pertinent questions and information needs of people at risk of T2D, with the individual, social and environmental determinants of eating identified as salient needs. Suggestions for how the design principles identified in Workshop 1 were discussed. Participants felt that mixed evidence or uncertainty surrounding specific elements of health advice should be identified to promote awareness and build trust. Language style was considered of critical importance with positive language & framing (e.g., use ‘within range’ rather than ‘good/bad’) and the use of icons to reduce words perceived as important strategies for communicating health information. These are summarised in Tables 2 and 3, which shows specific findings from the workshop, their links to persuasive design principles, and how they may be implemented within an intervention. This relates to the TDF domain, *environmental context and resources*. Desirable app features and characteristics included behaviour change support, such as meal planning strategies and recipes, fostering social connection with other users or professionals, and the ability to track and monitor their progress.

### Evaluation of prototypes

Figure 1 shows the four app concepts reviewed by participants in Workshop 3. Participants’ views on the *Choices (pathways and records)* concept informed three PD principles: (a) *self-monitoring*, (b) *praise*, and (c) *social learning*. This concept was perceived to provide a way of tracking users’ status or performance, supporting users in achieving their behavioural goals. Participants suggested that this concept should be complemented with a feature that provides users with positive feedback regardless of behaviour outcomes and allows users to “see other people journeys / choices / and adopt good practices”.

Some participants liked the *Caring (for a tree)* concept due to its visual appeal (PD principle: *liking*), however some participants thought that a tree lacked meaningful resemblance to humans (PD principle: *similarity*). On the other hand, *Caring (for an avatar)* was perceived by some participants to have meaningful resemblance to humans, although age-inappropriate for some.

Three PD principles were identified from participants’ perspectives on the *Competition (with self or others)* concept: (a) *self-monitoring*, (b) *competition*, and (c) *rewards*. Besides the ability to track progress, the ability to compete with other users was perceived as a motivator to adopt a target attitude or behaviour. However, it was recognised that unhealthy competition could lead to negative impacts. A suggestion for how this concept could be improved was to provide rewards to users as credit for working towards target behaviour goals.

### Participant feedback

Following each workshop, participants were asked to complete an anonymous feedback survey (mean response rate 73%). Feedback was positive; all respondents either *agreed* or *strongly agreed* that the activities were engaging and easy to follow. Several suggestions for improvement, such as having more than two participants per breakout group, were incorporated into subsequent workshops where possible. Participants also noticed the value of co-design, suggesting that the workshop activities and structures were appropriate and constructive. One participant stated in their feedback that “the importance of co-design is important but is often used a tick box ‘we have had the meeting with the community, but we don’t have to take any notice of what was said’, I felt that our views and lived experiences were seen as important (*in this co-design process*)”. For repeat participants, the continuity of activities and carry over of information/ideas between workshops was also appreciated. One participant mentioned one thing they liked about Workshop 2 was “Seeing the synthesis of our ideas and thoughts from Workshop 1.”

## Discussion

This study describes the co-design of a digital health promotion intervention for people at risk of T2D. Relevant app design principles identified included *social support* to help users connect with others, *reduction* to make performing target behaviour easier for users, and *tunnelling* to facilitate users’ access to tailored information. *Praise*, *rewards*, and *self-monitoring* were also frequently mentioned as desired app characteristics. The most preferred app prototype was the concept of *Choices (pathways and records)*, a concept allowing users to follow their individual journeys. Both researchers and designers agreed that the online co-design workshops were successful and participant feedback was highly positive.

### Comparison with the literature

This paper focuses on the diet aspects of T2D management as part of testing the application of the co-design approach in intervention development. Previous research has shown most digital dietary apps apply a combination of persuasive strategies to promote healthy eating among users [31], and our workshops’ findings closely align with most of these. Namely, *personalization* and *suggestion* (most dominant persuasive strategies), *self-monitoring*, *reduction*, *reminders*, *expertise*, *trustworthiness*, *surface credibility*, and *real-world feel*. The only persuasive strategy not identified was *commitment/consistency*, a strategy in which users commit to drinking only non-sweetened beverages every day, for example. Interestingly, the workshops uncovered several persuasive strategies which are less common in digital dietary apps [31]. These were *tunnelling*, *liking*, *similarity*, *rewards,* and *competition*. Inclusion and evaluation of these persuasive design elements in future digital health interventions warrant further investigation.

Findings from the co-design workshops add support to those of the formative Delphi study [24]. For example, in the Delphi study, end-users identified financial strain as a barrier to healthy habits. This issue was raised in Workshop 3; when asked what should be included in a T2D app, a participant stated: “Budget menu planner, with recipes, so if you’re broke you can still eat for good T2 management …” . It should be noted that such strategies and indeed, digital health more broadly, is not expected to be able to help users overcome social determinants of health including poverty and access to healthy foods. Instead, their utility may be limited to providing behaviour change support for individuals who are ready and able to undertake it. Notwithstanding, congruence between the co-design and Delphi study findings strengthen the overall co-design project as frequently raised matters can be leveraged in the intervention design.

In a previous co-design study that aimed to design an app to encourage physical activity among older adults [20], an app feature raised by participants was the ability to collect, record and share health data with healthcare professionals. In the present study’s workshops, similar discussions surrounded this feature; with a participant stating: “When trying to reach goals, or maintain a standard, to check in and see where we are at, to share with doctors, or community …” . Social connections, as well as rewards and encouraging messages, were two other commonly desired app features identified in both studies, although it should be noted that the use of broadband social media was not desirable. Each study also identified other app features unique to the purpose of the intervention being developed (i.e., physical activity versus diet). This highlights the possibility of identifying commonly desired app features despite differences in the health behaviour that each unique intervention aims to promote.

### Strengths and limitations

Overall, observations from the research and design team discussions combined with participant feedback indicated that the co-design process was successful, and the workshops produced insights and prototypes that the research team initially set out to establish.

#### Application of PD principles in app development

Previous literature suggests it is crucial to consider *how* PD principles are operationalised and presented as design features, as this will determine the “potential persuasive effectiveness” of an app in promoting behaviour change [30]. Furthermore, there is a lack of discussion in the literature of how PD principles could be applicable in the context of dietary behaviour change apps. A strength of this study is that PD principles were identified and translated into examples of actual app features and characteristics, providing guidance for design vision and strategies more likely to elicit behaviour change.

#### Online workshop facilitation

Although originally planned to be held in-person, the COVID-19 pandemic meant that activities needed to be shifted online. Fortunately, a number of online tools were available to facilitate this. However, there is an absence of literature reporting on the use of this format to perform co-design of health-related interventions. The present study suggests an online environment may be an appropriate, feasible and effective delivery approach for workshop planning and execution that provides increased efficiency and improves the typical time-intensive nature of co-design for the participant. However, it should be noted that there is still a substantial amount of preparatory and operational work required of the facilitators to deliver a smoothly run, online co-design workshop. Another benefit of the online format is that it makes it possible to overcome geographical or mobility barriers to participation that fostered inclusive research practices.

#### Design expertise

The utilisation of experienced facilitators trained in co-design combined with highly developed and unique skills of the designers enriched the research process [32].

#### Visual thinking

A unique characteristic of co-design is the application of visual thinking in workshop activities. In the third workshop, participants were asked to select desirable T2D app features from an array of abstract icons. It was the intention of the designers to use abstract icons, rather than specific detailed examples of existing interventions, for example, to indirectly generate a broad range of ideas from participants. This visual strategy can stimulate creativity and is designed to allow researchers and designers to understand the implicit meaning in participants’ engagement with the activity. In addition, the use of abstracted icons can assist in dissembling power structures such as assumed prior knowledge of health and medical stimulus.

#### Recruitment challenges

This study has several limitations. The target sample size was not met (participant attendance met 36–60% of the workshop attendance target), which may have been due at least in part to the online delivery of the workshops, the necessity to coordinate common times and the relative ease of not attending. Furthermore, it was not possible to ensure a consistent of different health status, educational attainment, or other demographic characteristics across the three workshops because participants were able to attend as few or as many workshops as desired. This is consistent with previous research and recruiting representative research samples is an ongoing challenge in public health [33]. Consequently, the proportions of people with T2D or people with higher/lower education varied between the workshops. This may have influenced the prioritisation and interventional features and characteristics identified that may potentially limited the generalisability of the findings. However, the replication of themes across multiple groups suggests the sample may have been large enough to establish a degree of saturation of perspectives. It was challenging to schedule a time which suited all interested participants; however, future co-design protocols researchers should attempt to avoid conducting workshops during late afternoon peak hours as per participant feedback. Health professionals were also underrepresented in the sample due to their lower proportion who attended the workshops. It is acknowledged that additional workshops may be needed to include participants in subsequent phases of the broader co-design project to increase generalisability of the findings and app. Nevertheless, a benefit of having fewer participants per workshop allowed for an increased depth of engagement with individual participants.

#### App as the pre-determined digital intervention

The funding for this project directed the co-design process toward the development of an app-based intervention. While this allowed the project team to focus the co-design process on the exploration of digital solutions, the dictation of this as a requirement limited the ability of the process to critically evaluate the assumption that an app-based solution was ideal. In particular, digital health excludes people who do not use the internet for health-related reasons. The digital divide captures that older adults, particularly those who experience other socio-economic disadvantage [34]. Further work is needed to bridge the digital divide and to design digital health interventions that are appropriate and appealing to older adults, as well as non-digital alternatives. The project team acknowledge the importance of establishing constraints, but the opportunity to question fundamental considerations such as this, may require a different understanding of the ways in which co-designed health behaviour projects are funded. It is also important to acknowledge there is no ‘one-size-fits-all’ solution and that an advantage of the co-design approach is that it can reveal and identify multiple factors that promote behaviour change that could be used to tailor interventions for personal preferences and needs.

### Implications for practice, policy and future research

The present study has implications for preventive health practice. The growing use of co-design to develop digital health interventions may help policymakers explore co-designed interventions as sustainable and person-centred disease prevention programs to address chronic disease burden. The present study provides a template for other researchers to ascertain feasibility in developing other health behaviour change apps. Formal evaluation of the effectiveness of digital health interventions developed using co-design methods is warranted.

### Directions for further work

This study only describes the first part of the co-design process. Due to the iterative nature of co-design, the input of end-users and key stakeholders remain crucial in the re-developments and concept refinement of the testable prototype to ensure the app is adaptive to the specific and evolving needs of end-users and other stakeholders. Engaging nutrition experts in this process is also necessary to curate information and content that are accurate, and appropriately facilitate a healthy behaviour change process [35]. Moreover, participation from nutrition experts would facilitate understanding of health professionals’ behaviours towards using technologies that will support incorporation of new technologies into dietetic professional practice to foster the digital health trend [36].

In developing a useable prototype, specific features to be incorporated into the app needs to be decided upon. This process could benefit from the more direct application of behaviour change techniques (BCTs) [37] to each feature, particularly BCTs that are associated with greater effectiveness [38]. The TDF domains attributed to the co-design workshop findings should be used to identify relevant BCTs.

In the long term, an important stage in the overall co-design project will be to ascertain the cost-effectiveness of the app which remains under-studied [39]. This will require rigorous evaluation in randomised controlled trials (for efficacy) as well as robust real-world community evaluations to understand wide-scale implementation potential. These stages are necessary to contribute to the development of a markedly accepted instrumental framework or theory of change that could explain and justify that co-design research methods lead to better health outcomes [39].

## Conclusion

This study demonstrated that co-design protocols is a feasible approach to understanding stakeholder needs and desired app features in developing a digital dietary intervention for adults at risk of T2D. It is acknowledged that this study has only examined the appeal of the digital intervention and thus conclusions about its efficacy in diabetes prevention cannot be made. Further work is needed to maintain a high level of engagement with end-users and stakeholders to develop final prototypes for real-world testing. Future research should also examine the use and effectiveness of co-design in developing digital dietary interventions in other relevant health domains.

## Supplementary Information

**Additional file 1.** Characteristics of Co-Design Workshop Participants.

## Data Availability

The datasets used and/or analysed during the current study are available from the corresponding author on reasonable request.

## Abbreviations

T2D: Type 2 diabetes
PD: Persuasive design
PAR: Participatory action research
TDF: Theoretical domains framework
