# Supplementary material for: Co-design of a digital dietary intervention for adults at risk of type 2 diabetes
Source: BMC Public Health. 2021 Nov 11;21:2071. doi: 10.1186/s12889-021-12102-y (PMC8582335; doi:10.1186/s12889-021-12102-y)
Supplement: Supplementary file 1 — Additional file 1. Characteristics of Co-Design Workshop Participants. [file 12889_2021_12102_MOESM1_ESM.docx]

**Additional file 1**

*Characteristics of Co-Design Workshop Participants*

| Characteristic | | Workshop 1  % (n) | Workshop 2  % (n) | Workshop 3  % (n) | Total ^a^  % (n) |
| --- | --- | --- | --- | --- | --- |
| Stakeholder type | End-user | 67 (8) | 78 (7) | 92 (11) | 83 (20) |
|  | Scientific/clinical experts | 33 (4) | 22 (2) | 83 (1) | 17 (4) |
|  | Total | N=12 | N=9 | N=12 | N=33 |
| Sex | Female | 83 (10) | 100 (9) | 58 (7) | 71 (17) |
| Age (years) | Median | 55 | 55 | 59.5 | 59 |
|  | Range | 38–63 | 38–61 | 40–62 | 38–63 |
| Highest level of education attained | Year 12 or equivalent | 8 (1) | 22 (2) | 42 (5) | 21 (5) |
|  | Trade certificate or diploma | 25 (3) | 33 (3) | 42 (5) | 38 (9) |
|  | University degree (e.g., bachelor’s degree) | 25 (3) | 11 (1) | 8 (1) | 17 (4) |
|  | Postgraduate university degree | 42 (5) | 33 (3) | 8 (1) | 25 (6) |
| Cultural identity | Australian | 83 (10) | 89 (8) | 75 (9) | 79 (19) |
|  | Chinese | 8 (1) | 11 (1) | 0 (0) | 8 (1) |
|  | English | 8 (1) | 0 (0) | 8 (1) | 17 (2) |
|  | New Zealand | 8 (1) | 0 (0) | 0 (0) | 8 (1) |
|  | South African | 0 (0) | 0 (0) | 8 (1) | 8 (1) |
|  | Irish | 0 (0) | 0 (0) | 8 (1) | 8 (1) |
|  | Scottish | 0 (0) | 0 (0) | 8 (1) | 8 (1) |
| Health status | Pre-diabetes | 25 (3) | 22 (2) | 17 (2) | 21 (5) |
|  | Type 2 diabetes | 8 (1) | 11 (1) | 67 (8) | 38 (9) |
|  | None of the above | 42 (5) | 44 (4) | 8 (1) | 25 (6) |
|  | Unsure | 8 (1) | 11 (1) | 8 (1) | 4 (1) |
|  | Did not indicate in survey | 17 (2) | 11 (1) | 0 (0) | 13 (3) |
|  | Has other serious health conditions | 8 (1) | 11 (1) | 0 (0) | 8 (2) |
| Index of disadvantage SEIFA ^b^ score by postal code residence | 1-25 (percentile) | 33 (4) | 22 (2) | 25 (3) | 25 (6) |
|  | 26-50 (percentile) | 25 (3) | 33 (3) | 33 (4) | 33 (8) |
|  | 51-75 (percentile) | 0 (0) | 22 (2) | 8 (1) | 8 (2) |
|  | 76-100 (percentile) | 42 (5) | 22 (2) | 33 (4) | 33 (8) |

^a^ Total number does not distinguish participants who attended more than one workshop.

^b^ SEIFA = Socio-Economic Indexes for Areas [30]. A lower score indicates that an area is relatively disadvantaged compared to an area with a higher score.
